# Heterogeneity of Treatment Outcomes Across Therapists and Sites in a Randomized Multicentre Psychotherapy Trial

**DOI:** 10.1002/cpp.70087

**Published:** 2025-05-21

**Authors:** Jonas Petter, Lea Schumacher, Jette Echterhoff, Jan Philipp Klein, Elisabeth Schramm, Martin Härter, Martin Hautzinger, Levente Kriston

**Affiliations:** ^1^ Department of Medical Psychology University Medical Center Hamburg‐Eppendorf Hamburg Germany; ^2^ Department of Psychiatry, Psychosomatics and Psychotherapy University of Lübeck Lübeck Germany; ^3^ Department of Psychiatry and Psychotherapy, Medical Center, Faculty of Medicine University of Freiburg Freiburg Germany; ^4^ Department of Psychology, Clinical Psychology, and Psychotherapy Eberhard Karls University of Tübingen Tübingen Germany

**Keywords:** clinic effect, psychotherapy, randomized controlled trial, study design, therapist effect

## Abstract

**Objective:**

Establishing robust evidence for psychotherapeutic treatment efficacy is crucial in evidence‐based medicine for mental disorders. Randomized controlled trials (RCTs) are key to minimizing biases such as selection effects and baseline imbalances between study groups. However, another challenge to robust evidence in psychotherapy research is heterogeneity in treatment outcomes due to therapists and clinical sites. While this has been frequently observed in naturalistic settings, therapist‐ and site‐related heterogeneity in treatment outcomes has been understudied in RCTs.

**Method:**

The present study addresses this gap in a secondary data analysis, examining how therapists and clinical sites differ in treatment outcomes and differential/average treatment effect (i.e., outcome differences between treatment groups) within a large, multicentre RCT. We analysed data from 255 patients with chronic depression treated by 79 therapists in nine study sites, that received two different active psychotherapeutic interventions.

**Results:**

Therapist‐ and site‐related variances in treatment outcomes appeared relatively small, accounting for 1.1% [0.0%, 8.1%] and 1.7% [0.0%, 9.9%] of the total variance, respectively. Notably, site‐related variance in differential treatment effects appeared relatively larger at 12.5% [0.1%, 44.4%]. These variances were only partially explained by patient or therapist characteristics.

**Conclusion:**

While the sample size only allowed to estimate the variance contributions of therapists and sites with high uncertainty, the relative size comparison points to the importance of considering site heterogeneity in evaluating RCTs' differential treatment effects. Further research on site characteristics' impact could enhance understanding of psychotherapeutic treatment efficacy across diverse contexts.

**Summary**

- This study highlights the importance of considering differences between treatment sites when evaluating psychotherapeutic treatments.
- This approach could make findings from psychotherapy studies more robust and, consequently, improve evidence‐based mental healthcare.
- Specific characteristics of therapists and clinical sites that relate to treatment outcome are difficult to establish.

Mental illness is a major public health concern, imposing suffering on the affected and a substantial economic burden on society (Insel 2008; Whiteford et al. 2015). Given the critical importance of addressing mental illness, considerable research effort and funding have been dedicated to evaluating the efficacy of psychological and other treatment approaches. However, establishing robust scientific evidence for psychotherapy to inform evidence‐based practice poses numerous challenges requiring carefully designed studies (Kazdin 2008; Loudon et al. 2015).

The field of experimental design, particularly in clinical trials, has been extensively studied and continues to evolve. Especially in recent years, rigorous experimental design has moved into focus, partly in response to the replication crisis in psychology (Pashler and Wagenmakers 2012). Experimental research in psychology is susceptible to various biases, many of which are now well recognized by researchers. Traditional biases include expectancy effects, observer biases and selection effects (Rosenthal 1976). Over time, researchers have refined their experimental designs to mitigate these biases, for instance, by implementing blinding techniques and randomizing participant assignment (Schulz and Grimes 2002). Consequently, psychotherapy research has established the randomized controlled trial (RCT) as the gold standard for clinical evidence (Hoffmann et al. 2014)—although this assertion rests on many, sometimes strong assumptions (Cook 2018). RCTs are designed to minimize the aforementioned biases, particularly those involving researchers and participants. However, psychotherapy research faces another set of unique challenges, absent from most other clinical trials: the potential heterogeneity introduced by different therapists and clinical settings. Addressing this challenge of heterogeneity in treatment outcomes between therapists and sites within RCTs is the central focus of the present study.

Outside of clinical trials, the question of variance in treatment outcome between therapists and clinical settings has long been of interest (Ricks 1974). Intuitively and anecdotally, patients and therapists alike are aware that some therapists and clinical institutions systematically achieve better outcomes than others. However, only more recently large amounts of routinely collected outcome data became available, providing the necessary resources to investigate this question in detail (Deisenhofer et al. 2024; Firth et al. 2019; Janse et al. 2024; Johns et al. 2019; Mahon et al. 2023; Pybis et al. 2017). These studies have shown that there is indeed a non‐trivial amount of heterogeneity in treatment outcome attributable to therapists and clinical sites. Which characteristics explain the heterogeneity between therapists remains an open question. Thus far, no therapist characteristics could be established as robust predictors of treatment outcome, including therapist experience levels or allegiance to any specific therapeutic approach (Baldwin and Imel 2013; Wampold and Owen 2021). Similarly, there is no decisive evidence regarding clinical institutions' characteristics that relate to treatment outcomes; however, studies have shown that the socio‐economic profile of the institutions' location (e.g., median income; Firth et al. 2019) and the organizational culture (Falkenström et al. 2018) both potentially play a role.

Up until now, most studies investigating therapist‐ and site‐related heterogeneity have been conducted in naturalistic settings, as opposed to investigating this question in RCTs. A meta‐analysis by Baldwin and Imel (2013) on therapist‐related outcome variance indicated less therapist‐related variance in RCTs compared to naturalistic settings. On the other hand, a smaller but more recent review pointed in the opposite direction (Johns et al. 2019). A smaller therapist‐related outcome variance in RCTs compared to naturalistic settings would be expected, as many potential sources of variance between therapists should be accounted for through standard elements of well‐designed RCTs, that is, randomization of patients, strict supervision, (continuous) adherence testing, reliance on treatment manuals and standardized protocols for several other processes.

There are only few studies that explicitly studied site‐related outcome variance in RCTs. Vaezazizi et al. (2019) showed that 6.7% of treatment outcome of an intervention for substance use disorder was accounted for by the treatment site. However, the investigated intervention was technology‐based and hence did not include any variance due to therapists, making an inference to trials including therapist‐specific variance difficult. Other RCTs, investigating psychotherapeutic interventions, have controlled for site variance in their statistical analysis (e.g., Bickman et al. 2011) but often without reporting the magnitude of this variance (e.g., Nakimuli‐Mpungu et al. 2020). However, due to the more controlled setting, a smaller variance in outcome between sites in RCTs compared to a naturalistic setting also appears intuitive. Furthermore, multicentre RCTs in which all sites provide multiple study treatments allow investigating a unique source of variance: the variance in the average treatment effect. To emphasize the distinction from the treatment outcome (i.e., the average outcome across both treatments), we decided to use the less commonly used term *differential treatment effect*. Variance in the differential treatment effect means it can be investigated if the difference in treatment outcome of two or more distinct treatments varies across study centres. Variance in differential treatment effect is usually only observable across sites, as therapists rarely administer multiple treatments in a RCT. While we could not detect any research dedicated explicitly to studying this potential variance in differential treatment effect, it should be also small, as study centres in multi‐site trials follow standardized protocols, minimizing potential biases, for example, through differences in implementation. Still, site‐related heterogeneity in differential treatment effects should be assessed, as it potentially introduces biases into RCTs that might necessitate controlling for beyond randomization.

In sum, the variance attributable to different therapists and sites in the presumably highly controlled setting of RCTs has been relatively underexplored. To fill this gap, the present study aims to answer two questions. First, we aimed to estimate the amount of site‐ and therapist‐related heterogeneity in a well‐designed, large psychotherapy RCT. This could give us insight into whether these sources of heterogeneity can be controlled for through experimental design. This information can help developing experimental designs further, to potentially reduce these forms of bias or indicate the necessity to control for these effects in statistical analyses. Second, we aimed to investigate if controlling for certain patient and therapist characteristics explains parts of the therapist‐ and site‐related variance. This could give us insight into their aetiology, which has the potential to identify factors for optimizing treatment outcome. To answer these questions, we conducted a secondary analysis of a multicentre psychotherapy RCT investigating heterogeneity of treatment outcomes across therapists and sites, heterogeneity of the differential treatment effect between sites, and potential patient and therapist characteristics that may explain this heterogeneity.

## 1 Methods

### 1.1 Procedure and Participants

We analysed data from a multi‐site, assessor‐blind, prospective, parallel‐group psychotherapy RCT. The study focused on assessing the impact of two active interventions, Cognitive Behavioral Analysis System of Psychotherapy (CBASP) and supportive psychotherapy (SP), in patients diagnosed with early‐onset chronic depression. Methodological details are documented in prior publications (Schramm et al. 2011, 2017) and are only summarized here. Approval for the study was granted by the Institutional Ethical Committee of the University of Freiburg, Germany, along with all other participating institutions. The RCT was registered with ClinicalTrials.gov (NCT00970437), and the present secondary data analysis was preregistered on the Open Science Framework (https://osf.io/9rvfx/?view_only=8f03413c8c1a4e68bc90d19441d73bca). A few minor deviations from the preregistered analysis are listed in the Supporting Information. Recruitment for the study took place across nine sites in Germany, spanning from 5 March 2010 to 16 October 2012. All patients were treated at the site where they initially presented. Each site administered both treatments, with patient randomization to treatment groups stratified across sites. Patients were assigned to therapists according to routine care procedures at each site (which were not documented), and each therapist delivered only one intervention, as determined by the site's principal investigator. The therapists received manualized and supervised training in the respective intervention. Each intervention included 20 weeks of acute treatment encompassing 24 individual sessions, followed by a continuation phase lasting 28 weeks with eight sessions in each treatment arm.

The CBASP intervention was structured and manualized. It is the only psychotherapy approach developed specifically for chronic forms of early‐onset depression and based on modern theories of interpersonal learning (McCullough 2003). That is, CBASP focuses on using the therapeutic relationship to target dysfunctional interpersonal behaviour. The SP intervention adopted a non‐specific but manualized approach and served as the active control condition. It is comparable to clinical management or client‐centred counselling (Markowitz 2022; Markowitz et al. 2008). SP focuses on non‐specific therapeutic elements such as interest, concern and understanding.

Assessments of depression symptoms and other related variables were conducted prior to treatment initiation (t0) and subsequently after 12 (t1), 20 (t2) and 48 weeks (t3). The present analysis focuses on data pertaining to depression symptoms measured at t0 and t2 and potential covariates measured at t0, where t0 marks the baseline measurement and t2 marks the end of the acute treatment phase, the primary measurement point of the original trial.

### 1.2 Measured Variables

#### 1.2.1 Therapist Characteristics

Demographic data, including age and gender, were obtained from participating therapists. Furthermore, they were asked to fill out a self‐report questionnaire to indicate their academic background (i.e., medicine or psychology), their total experience administering psychotherapy in months, the number of previously completed cases administering the study‐relevant treatment (i.e., CBASP or SP), if they had previous experience with manualized therapy, as well as their attitudes towards the assigned treatment, measured with a self‐designed questionnaire, ranging from 0 (*negative attitude*) to 15 (*positive attitude*).

#### 1.2.2 Patient Characteristics

General demographic information, including age, gender and income, and the preference for psychotherapy or medication were collected from the patients. Their medical records were consulted to obtain information about previous or present medical disorders, outpatient and/or inpatient psychiatric and/or psychotherapeutic treatments and past suicide attempts.

#### 1.2.3 Psychopathology

A range of psychological measurements were conducted. Depression symptoms were assessed using the 24‐item version of the Hamilton Rating Scale of Depression (HDRS; Hamilton 1960). Anxiety levels were measured using the General Anxiety Disorder Assessment (GAD‐7; Spitzer et al. 2006). Interpersonal problems were measured with the 64‐item self‐report Inventory of Interpersonal Problems (IIP‐64; Horowitz et al. 1988). Social functioning was measured by the Social Adaptation Self‐Evaluation Scale (SASS; Duschek et al. 2003). Childhood trauma was measured by the Childhood Trauma Questionnaire (CTQ; Bernstein et al. 1994). Suicidal ideation was measured with the Beck's Scale for Suicidal Ideation (BSSI; Beck et al. 1988).

### 1.3 Statistical Analysis

To gain a detailed understanding of the variance in treatment outcomes and the differential treatment effects, we compared statistical models that incorporated different predictors. The treatment outcome was defined as the difference between depression severity at t0 (baseline) and t2 (20 weeks), measured in HDRS sum scores. This constituted the outcome variable for all models. In line with the preregistration, we fitted Bayesian three‐level multilevel models, with data from patients (Level 1), nested within therapists (Level 2), who were nested in study sites (Level 3). As the sample size and number of clusters appears insufficient to precisely estimate the random effects at all three levels with traditional maximum likelihood–based approaches (Maas and Hox 2005), we opted for a Bayesian approach allowing for a clear quantification of this uncertainty. Furthermore, Bayesian estimation has been shown to perform generally more reliably than frequentist methods with small samples (Baldwin and Fellingham 2013). All models included random intercepts for therapists and sites and a random slope for treatment group on the site‐level, that is, the differential treatment effect. This means, in all models, the treatment outcome (HDRS reduction) was allowed to differ between therapists and study sites, and the differential treatment effect of CBASP versus SP could differ between sites. This model specification allows us to investigate the heterogeneity in treatment outcomes between therapists and sites, as well as the heterogeneity in differential treatment effect between sites. As no therapist administered both treatments, we cannot compute a random slope for treatment groups at the therapist level.

To quantify the variance attributable to therapists and study sites, we calculated the variance partition coefficient (VPC; e.g., Lewis et al. 2010). The VPC describes the percentage of the total variance in an outcome that can be attributed to differences across the hierarchical levels. Formally, it is expressed as in Equation (1).

(1) $$\mathrm{VPC}=\frac{\sigma_{\text{group}}^{2}}{\sigma_{\text{total}}^{2}},$$

where $\sigma_{\text{group}}^{2}$ denotes the estimated random effect for an intercept/slope and $\sigma_{\text{total}}^{2}$ the estimated total variance in the model.

We investigated (1) the VPC for the treatment outcome between therapists (therapist random intercept), (2) the VPC for the treatment outcome between sites (site random intercept), and (3) the VPC of the differential treatment effect between sites (site random slope). To allow a comparison of the relative sizes of these VPC, we assessed the proportion of posterior samples in which each variance is the largest variance, excluding the residuals from the comparison.

In total, we assessed five models, each comprising different predictors. First, to answer the question regarding the amount of site‐ and therapist‐related heterogeneity that remains in a well‐designed, large psychotherapy RCT, we estimated a *variance partitioning model* to estimate the variance attributable to differences between therapists and study sites. This model contained only the treatment group (CBASP/SP) and baseline depression scores (measured with the HDRS) as predictors. The formal expression of the variance partitioning model is given in (2), with the remaining models detailed in the Supporting Information.

(2) $$\begin{array}{c} y_{\mathrm{ijk}}=\beta_{000}+u_{00k}+u_{0\mathrm{jk}}+\left(\beta_{100}+u_{10k}\right){\text{Treatment}}_{\mathrm{ijk}}\\ +\beta_{200}{\text{BaselineDep}}_{\mathrm{ijk}}+e_{\mathrm{ijk}}, \end{array}$$

where $y_{\mathrm{ijk}}$ represents the outcome for patient *i*, with therapist *j*, in clinic *k*. The intercept term $\beta_{000}$ is the overall mean, while $u_{00k}$ is the random intercept at the clinic level. The term $u_{0\mathrm{jk}}$ represents the random intercept at the therapist level. The coefficient $\beta_{100}$ corresponds to the overall treatment effect, with $u_{10k}$ representing the random slope for treatment at the clinic level. ${\text{Treatment}}_{\mathrm{ijk}}$ is the treatment indicator, and ${\text{BaselineDep}}_{\mathrm{ijk}}$ represents the baseline depression score for patient *i*. Finally, $e_{\mathrm{ijk}}$ is the residual error at the patient level.

Next, we assess models with additional covariates, to investigate if controlling for certain patient and therapist characteristics explains parts of the therapist‐ and site‐related variance. Our second model is the *therapist characteristics model*, which also included therapist characteristics as covariates. Therapist characteristics pertains to all variables listed in the previous section under therapist characteristics. Third, a *patient characteristics model* was estimated, that is, the variance partitioning model additionally including the patient characteristics as covariates. Patient characteristics pertains to all variables listed in the previous section under patient characteristics and psychopathology. Fourth, a *patient and therapist characteristics model* was estimated, with both patient and therapist characteristics included as covariates. Lastly, we estimated an *extended patient and therapist characteristics model*, that is, the patient and therapist characteristics model including measures of congruence between patients and therapist characteristics as covariates. The measures of congruence between patients and therapists were calculated as the similarity in age and gender, as these are the only variables available for both patients and therapists. Table 1 summarizes the five models and their components.

**TABLE 1:** The investigated models and their predictor components.

Model	Design components	Therapist characteristics	Patient characteristics	Congruence
Variance partitioning model	✓	✗	✗	✗
Therapist characteristics model	✓	✓	✗	✗
Patient characteristics model	✓	✗	✓	✗
Patient and therapist characteristics model	✓	✓	✓	✗
Extended patient and therapist characteristics model	✓	✓	✓	✓

*Note:* Design components refer to the treatment group and the baseline depression score. Congruence refers to the similarity between therapists and their patients, based on age and gender.

As a secondary outcome measure, we also estimated five models with the number of attended therapy sessions as outcome and the same predictors as listed above.

We fitted the models using R4.4.1 and its package *brms* (Bürkner 2017; R Core Team 2024). We imputed missing values using data augmentation, as implemented in the R package *mix* (Schafer and L. 2024). All predictors were grand‐mean centred, to ease the interpretation of coefficients, while preserving the influence of Level 1 predictors on the intercept variance (Enders and Tofighi 2007). Centring also simplifies the calculation of the VPC by allowing the random slopes to be modelled at their average level, thereby fixing their influence on the intercept variance at this average level (Snijders and Bosker 2011). We specified weakly informed prior distributions for the models, taking into account the differences in measurement scales between the predictors (see preregistration for the exact priors). This choice helped avoid strong assumptions, given the variability in findings across related studies and the exploratory nature of the current analysis. Posterior parameter distributions were estimated using Hamiltonian Monte Carlo sampling from the joint posterior. Convergence of the sampler was assessed through the R‐hat statistic (Gelman and Rubin 1992) with a cut‐off below 1.01 and by visually inspecting the trace plots. The analysis code is publicly available on the Open Science Framework (https://osf.io/xuhmb/?view_only=9d08da2f92db4f86892d8e8593867c62).

## 2 Results

### 2.1 Descriptive Analyses

An overview of the patient and therapist characteristics is displayed in Tables 2 and 3, respectively. In total, 255 patients, treated by 79 therapists across nine treatment sites were included in the analysis. The patients were on average 44.9 years (SD = 11.8) old and identified mostly as female (66%). Their average depression severity at baseline measured with the HDRS was 27.19 (SD = 5.68), indicating severe depression. The therapists were on average 37.9 years (SD = 9.0) old and also mostly female (71%). They had on average, 79.4 months (SD = 64.2) of clinical experience. On average, 28.3 patients (SD = 12.2) and 8.8 therapists (SD = 2.5) participated in each study site. In each of the nine sites, between six and 14 therapists treated between one and 10 patients. Across clinic, each therapist treated on average 3.2 patients (SD = 2.1). There were only a small number of missing values across the variables, ranging from 0% to 11% of the total observations. A detailed overview of missing values per variable is presented in the Supporting Information.

**TABLE 2:** Descriptive statistics of the patients.

Variables	Total (*N* = 255)	Site 1 (*n* = 18)	Site 2 (*n* = 20)	Site 3 (*n* = 40)	Site 4 (*n* = 42)	Site 5 (*n* = 25)	Site 6 (*n* = 13)	Site 7 (*n* = 17)	Site 8 (*n* = 45)	Site 9 (*n* = 35)
Age	44.9 (11.8)	43.2 (12.0)	41.9 (10.6)	43.8 (12.9)	48.9 (10.6)	46.4 (8.1)	44.2 (10.4)	47.0 (10.0)	47.2 (12.7)	39.5 (13.3)
Gender (female)	66%	78%	55%	68%	55%	76%	69%	65%	76%	57%
Treatment preference (psychotherapy only)	76%	83%	65%	65%	90%	80%	77%	82%	58%	89%
Income (0–24)^a^	12.0 (6.0)	14.2 (5.9)	9.6 (5.9)	10.8 (6.1)	12.9 (5.6)	10.4 (5.7)	14.4 (5.5)	13.9 (4.8)	13.0 (6.6)	10.5 (5.8)
Depression baseline (HDRS; 0–75)	27.2 (5.7)	27.7 (4.6)	27.1 (5.1)	29.9 (5.8)	26.6 (4.9)	29.0 (6.6)	24.6 (6.4)	29.2 (5.8)	25.6 (5.8)	25.3 (4.6)
Anxiety Baseline (GAD‐7; 0–21)	10.9 (4.6)	12.2 (4.4)	9.6 (4.6)	12.9 (4.8)	11.0 (3.9)	11.2 (5.0)	9.0 (3.9)	10.4 (3.3)	9.9 (4.9)	10.2 (4.3)
Suicidality (BSSI; 0–38)	6.6 (7.3)	3.8 (4.6)	4.3 (5.5)	7.0 (7.6)	7.8 (7.7)	7.6 (8.0)	10.9 (9.3)	2.9 (4.1)	7.7 (7.9)	5.2 (6.4)
Social functioning (SASS; 0–60)	30.1 (6.5)	30.1 (6.7)	31.2 (6.4)	30.0 (7.7)	29.1 (6.4)	29.2 (6.7)	30.9 (6.8)	27.7 (6.0)	31.5 (6.1)	30.5 (5.1)
Interpersonal problems (IIP‐64; 0–256)	14.4 (3.8)	15.8 (3.3)	13.9 (4.1)	15.2 (4.3)	15.0 (3.6)	15.0 (2.4)	14.1 (4.2)	15.1 (3.0)	14.0 (3.8)	12.7 (3.8)
Childhood trauma (CTQ; 25–125)	52.8 (15.9)	50.4 (17.7)	47.9 (15.2)	54.0 (17.0)	56.6 (18.3)	57.8 (14.9)	55.7 (16.6)	52.7 (16.0)	52.7 (13.9)	46.4 (11.9)
Depression type (double depression)	45%	83%	40%	35%	50%	40%	38%	53%	58%	23%
Psychological comorbidities (at least one)	67%	83%	95%	69%	62%	72%	54%	88%	47%	63%
Treatment history (no previous treatment)	22%	28%	25%	15%	10%	16%	8%	35%	20%	46%

*Note:* All statistics that are not percentages are means with standard deviations in brackets.

Abbreviations: BSSI = Beck Scale for Suicide Ideation, CTQ = Childhood Trauma Questionnaire, GAD‐7 = Generalized Anxiety Disorder Scale, HDRS = Hamilton Rating Scale for Depression, IIP‐64 = Inventory of Interpersonal Problems (64‐item version), SASS = Social Adaptation Self‐Evaluation Scale.

^a^ Income was measured on a scale ranging from 0 to 24, where a 1‐point increase corresponds to approx. 250€ more per month.

**TABLE 3:** Descriptive statistics of the therapists.

Variables	Total (*N* = 79)	Site 1 (*n* = 6)	Site 2 (*n* = 7)	Site 3 (*n* = 7)	Site 4 (*n* = 11)	Site 5 (*n* = 8)	Site 6 (*n* = 7)	Site 7 (*n* = 9)	Site 8 (*n* = 14)	Site 9 (*n* = 10)
Age	37.9 (9.0)	45.2 (9.3)	36.7 (6.8)	37.4 (8.8)	35.4 (7.3)	34.6 (4.1)	36.6 (9.5)	40.1 (10.4)	36.4 (9.4)	32.2 (6.4)
Gender (female)	71%	50%	71%	86%	55%	62%	71%	67%	93%	70%
Academic background (psychology)	76%	50%	57%	100%	88%	13%	92%	44%	29%	100%
Clinical experience (in months)	79.4 (64.2)	103.0 (72.6)	61.3 (50.9)	74.0 (47.4)	72.4 (75.9)	74.6 (52.4)	92.0 (101.6)	94.7 (55.7)	68.9 (61.4)	48.1 (57.2)
Any previous experience with manualized treatments	63%	67%	29%	100%	55%	50%	71%	78%	50%	60%
Previous experience with assigned treatment (completed cases)	2.7 (5.7)	4.8 (8.1)	2.9 (6.7)	2.1 (4.5)	0.6 (1.5)	4.8 (8.9)	1.0 (1.9)	0.3 (0.7)	3.4 (7.4)	1.1 (2.0)
Assigned treatment attitude^a^ (0–15)	11.6 (1.9)	11.3 (3.0)	12.7 (1.6)	10.8 (1.2)	12.0 (1.8)	11.7 (2.1)	11.4 (2.4)	12.2 (1.8)	11.0 (2.3)	11.4 (1.3)

*Note:* All statistics that are not percentages are means with standard deviations in brackets.

^a^ A self‐designed questionnaire was used, with 0 indicating very negative attitudes and 15 very positive attitudes.

### 2.2 Variance Partitioning Model: Variance Attributable to Therapists and Sites

The variance partitioning model, that accounts only for the treatment group and baseline depression severity, indicated a small variance in the treatment outcome between therapists and study sites, as summarized in Table 4. Specifically, 1.1% and 1.7% of the total variance in the treatment outcome were attributable to differences between therapists and sites, respectively. The relative variance of the differential treatment effect between the sites was considerably larger and accounted for 12.5% of the total variance. In terms of the original measurement scale, this corresponds to an estimated standard deviation of approximately 1.0 points on the HDRS between therapists, 1.2 points between sites and 3.4 points for the differential treatment effect across sites. These values reflect the average spread in outcomes attributable to each source of variation. However, the 95% credible intervals indicate that these estimates contain a large amount of uncertainty. The absolute size of the variances is, thus, difficult to interpret. Still, comparing their relative size, the variance of the differential treatment effect between sites appears to be consistently the largest source of variance, as signified by the fact that it is the largest source of variance in 82.3% of the posterior draws (ignoring residuals).

**TABLE 4:** Summary of the variance partition for each model.

Variance source	Variance partitioning model		Therapist characteristics model		Patient characteristics model		Patient and therapist characteristics model		Extended patient and therapist characteristics model	
	Variance	VPC	Variance	VPC	Variance	VPC	Variance	VPC	Variance	VPC
Variance in treatment outcome between therapists	1.03 [0.00, 7.13]	1.1% [0.0%, 8.1%]	1.42 [0.00, 10.07]	1.6% [0.0%, 10.9%]	0.83 [0.00, 6.01]	1.0% [0.0%, 7.3%]	1.09 [0.00, 8.05]	1.4% [0.0%, 9.5%]	0.98 [0.00, 7.56]	1.2% [0.0%, 9.2%]
Variance in treatment outcome between sites	1.53 [0.00, 10.21]	1.7% [0.0%, 9.9%]	2.00 [0.00, 13.90]	2.3% [0.0%, 13.5%]	2.70 [0.02, 14.62]	3.4% [0.0%, 14.8%]	3.44 [0.03, 21.69]	4.4% [0.0%, 21.2%]	3.77 [0.03, 19.55]	4.7% [0.0%, 19.6%]
Variance in the differential treatment effect	11.33 [0.09, 62.79]	12.5% [0.1%, 44.4%]	7.38 [0.01, 49.50]	8.4% [0.0%, 38.2%]	10.00 [0.20, 56.99]	12.6% [0.3%, 44.3%]	7.55 [0.04, 48.17]	9.6% [0.1%, 39.6%]	8.02 [0.05, 48.44]	10.1% [0.1%, 39.9%]
Residual	76.41 [64.02, 92.12]	84.6% [52.9%, 97.8%]	77.40 [64.72, 92.60]	87.8% [57.2%, 98.5%]	66.14 [55.17, 79.26]	83.0% [51.4%, 96.6%]	66.56 [54.98, 81.25]	84.6 [53.0%, 96.9%]	66.89 [55.26, 80.84]	84.0% [52.3%, 96.9%]
Total	90.29 [72.21, 145.76]	100%	88.20 [72.38, 134.85]	100%	79.67 [63.82, 130.98]	100%	78.64 [63.74, 128.63]	100%	79.65 [64.00, 126.37]	100%

Abbreviation: VPC = variance partition coefficient.

**FIGURE 1:**
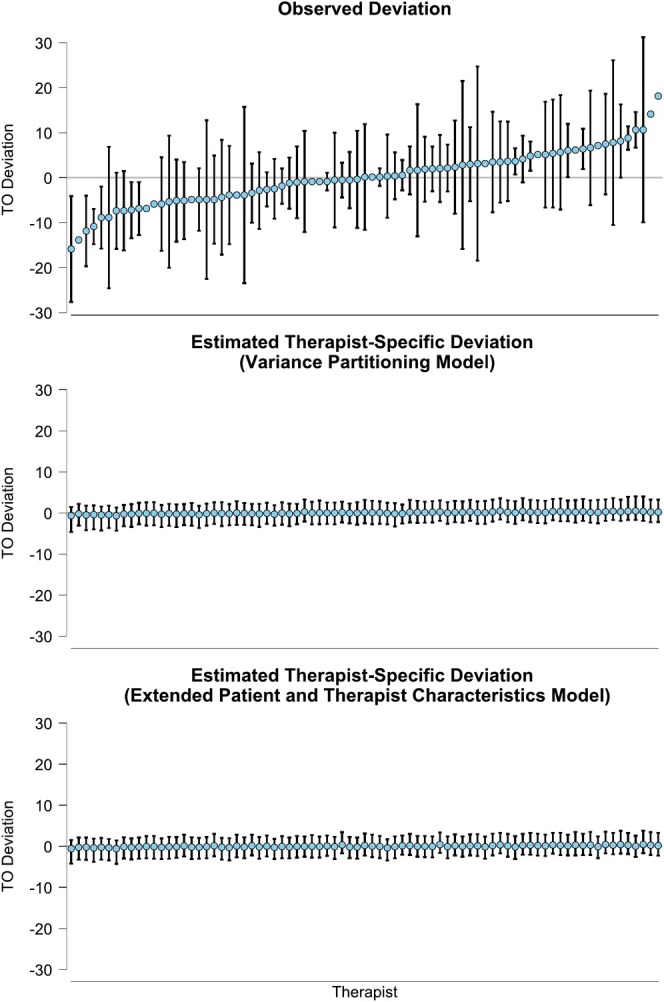
The deviation from the mean treatment outcome per therapist. *Note:* TO Deviation = Treatment outcome deviation, that is, the difference between the outcome of each therapist and the grand‐mean outcome across all therapists. Comparing the variance in deviation from top to bottom, we can see how much of the variance is explained through adding the respective predictors. The error bars show 95% asymptotic confidence intervals for the observed deviation and 95% credible intervals for the estimated deviation.

**FIGURE 2:**
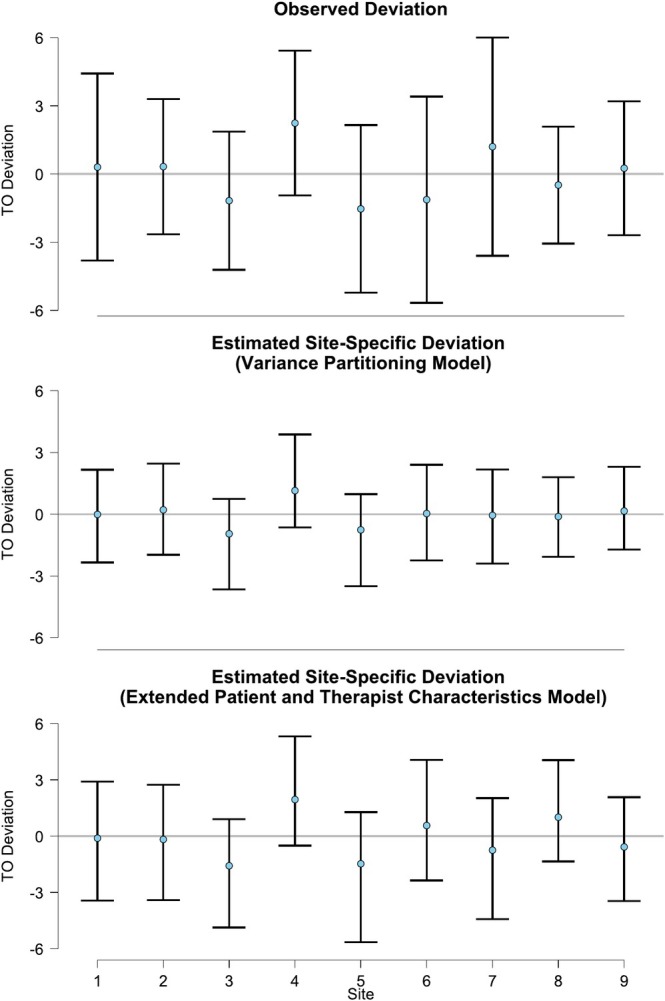
The deviation from the mean treatment outcome per site. *Note:* TO Deviation = Treatment outcome deviation, that is, the difference between the outcome of each site and the grand‐mean outcome across all sites. Comparing the variance in deviation from top to bottom, we can see how much of the variance is explained through adding the respective predictors. The error bars show 95% asymptotic confidence intervals for the observed deviation and 95% credible intervals for the estimated deviation.

**FIGURE 3:**
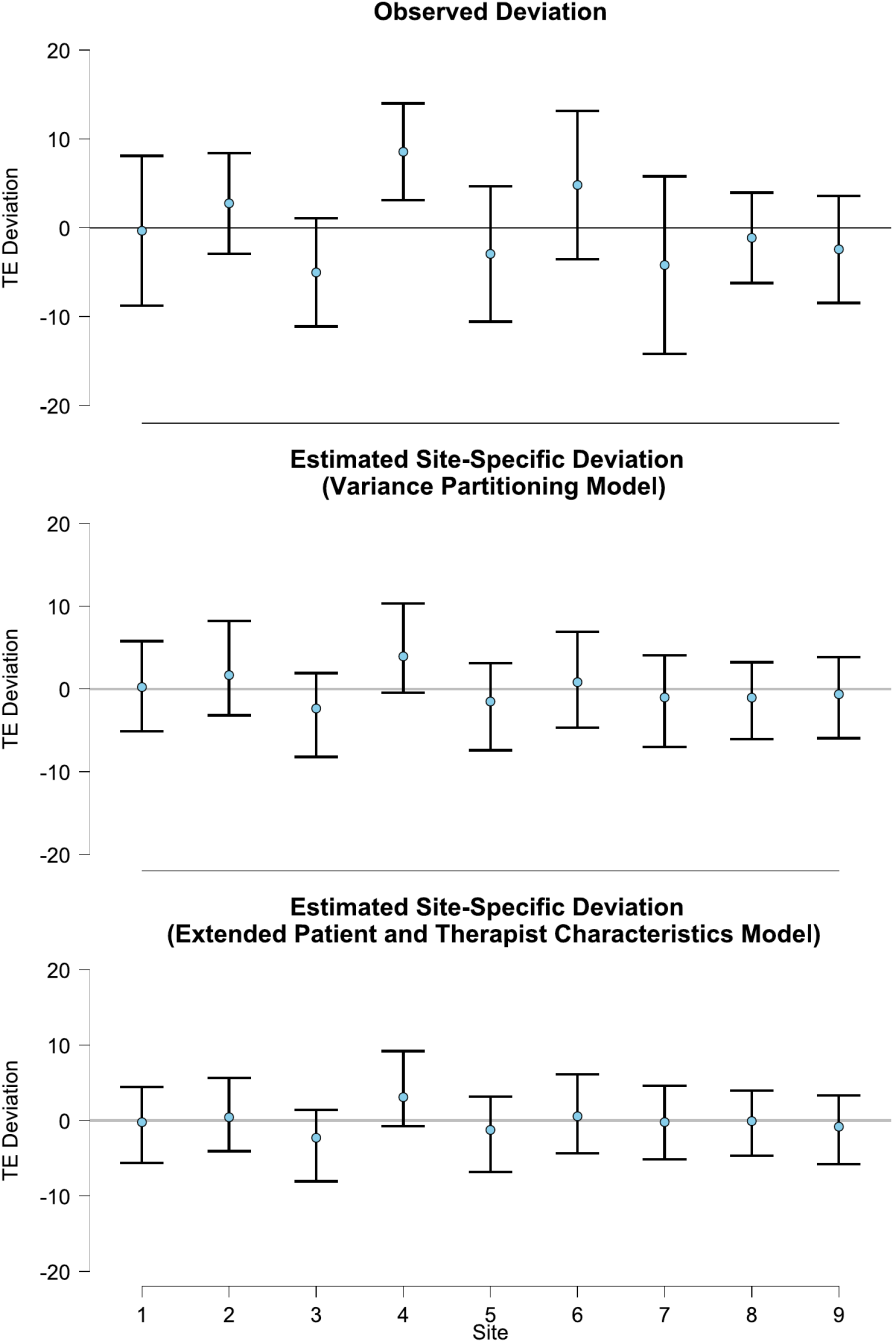
The deviation from the mean differential treatment effect per site. *Note:* TE Deviation = Treatment effect deviation, that is, the difference between the differential treatment effect of each site and the mean differential treatment effect across all sites. Comparing the variance in deviation from top to bottom, we can see how much of the variance is explained through adding the respective predictors. The error bars show 95% asymptotic confidence intervals for the observed deviation and 95% credible intervals for the estimated deviation.

The observed and estimated variances in treatment outcome between therapists and sites are also illustrated in Figures 1 and 2, respectively. The observed and estimated variances in differential treatment effect between sites are illustrated in Figure 3. For all sources of variance, we can see that the variance in the treatment outcome observed in the data (top panels) was larger than the variance attributable to therapists and sites as estimated from the variance partitioning model (middle panel). This indicates that the treatment group and baseline depression severity explain a large portion of the observed variance in treatment outcome.

### 2.3 Covariate Models: Explaining the Variance Between Therapists and Study Sites

The results of adding different blocks of predictors to the variance partitioning model are presented in Table 4. The median posterior estimates of variance components are reported below; however, the corresponding credible intervals were wide in all models. Therefore, these changes in explained variance should be interpreted with caution.

First, adding therapist characteristics to the model (therapist characteristics model) barely increased the relative outcome variance attributable to therapists from 1.1% to 1.6%, and the variance attributable to study sites from 1.7% to 2.3% compared to the variance partitioning model. The variance of the differential treatment effect between sites decreased from 12.5% to 8.4% of the total variance.

Second, adding patient characteristics to the model (patient characteristics model) marginally decreased the relative outcome variance attributable to therapists from 1.1% to 1.0%, and increased the relative variance attributable to sites from 1.7% to 3.4%, compared to the variance partitioning model. The relative variance of the differential treatment effect attributable sites increased marginally from 12.5% to 12.6%.

Third, adding both the patient and therapist characteristics to the model (patient and therapist characteristics model) barely increased the relative outcome variance between therapists from 1.1% to 1.4%, and the relative variance between sites from 1.7% to 4.4%, compared to the variance partitioning model. The relative variance of the differential treatment effect attributable to sites decreased from 12.5% to 9.6%.

Lastly, we added the patient and therapist characteristics, as well as the match in age and gender between patients and therapists to the model (extended patient and therapist characteristics model). This marginally increased the relative outcome variance attributable to therapists from 1.1% to 1.2%, and the relative variance attributable to sites from 1.7% to 4.7%, compared to the variance partitioning model. The relative variance of the differential treatment effect between sites decreased from 12.5% to 10.1%.

In sum, regarding the heterogeneity in treatment outcome attributable to therapists, none of the blocks of predictor variables explained a notable amount. Regarding the heterogeneity in treatment outcome attributable to sites, it appears that, if anything, it increases when taking the patient and therapist characteristics or the match between therapists and their patients into account. Lastly, regarding the heterogeneity in differential treatment effect attributable to study sites, all blocks of predictors but the patient characteristics appear to explain a some of the relative variance. Most notably, adding only the therapist characteristics reduces the relative variance in differential treatment effect between sites by about one third (from 12.5% to 8.4%). This is also visible in Figures 1, 2, 3, as the variance in the treatment outcome between therapists (Figure 1) and clinics (Figure 2) does not reduce when adding the patient and therapist characteristics (comparing the middle and bottom panels). However, the variance in the differential treatment effect attributable to sites does reduce slightly, as is visible when comparing the middle and bottom panel in Figure 3. This is further supported by the model comparison using leave‐one‐out cross‐validation (LOO‐CV) and WAIC, where the model including patient characteristics achieved the best overall predictive fit. Details of the model comparison are presented in the Supporting Information.

Again, similar to the variance partitioning model, the 95% credible intervals indicate a large amount of uncertainty in the variance estimates. Still, when comparing the relative size of the variances, the percentage of posterior draws in which the variance of the differential treatment effects between sites is the largest variance (ignoring the residual) decreases from 82.3% and 80.5% to 64.4%, 62.8%, and 64.0% in models without and with therapist characteristics included as covariates, respectively. Standardized regression coefficients for the individual patient and therapist characteristics included in each model were generally small, ranging from 0.01 to 0.16, and are reported in the Supporting Information.

### 2.4 Secondary Outcome: Number of Therapy Sessions

Investigating the number of attended therapy sessions as an alternative outcome measure led to similar results. In the variance partitioning model, the relative variance of the outcome between therapists and sites were small, with 0.8% and 1.4%, respectively. The relative variance of the difference between the two treatment groups was larger, with 11.5% of the total variance. The small relative variances between therapists and sites were not reduced further when adding any of the predictor blocks. The relative variance of the difference between the two treatment groups reduced most strongly in the therapist characteristics model, where it accounted for 9.6% of the total variance. Detailed results for the models with the number of therapy sessions as outcome are presented in the Supporting Information.

## 3 Discussion

Previous research has demonstrated that treatment outcomes can vary significantly between therapists and treatment centres (Deisenhofer et al. 2024; Firth et al. 2019; Janse et al. 2024; Johns et al. 2019; Mahon et al. 2023; Pybis et al. 2017). RCTs usually take measures aimed at reducing this heterogeneity, such as randomization and the use of standardized treatment protocols. However, the extent of therapist‐ and site‐related outcome heterogeneity that remains despite these measures and the factors contributing to this heterogeneity are not well understood. To address these gaps, we examined the therapist‐ and site‐related heterogeneity in treatment outcomes and site‐related heterogeneity in the differential treatment effect in a large, multicentre psychotherapy RCT. Additionally, we investigated factors that might explain this heterogeneity.

Our analyses showed that the variance in treatment outcome between therapists and study sites was comparatively small, that is, treatment outcomes for patients of different therapists and sites did not differ strongly when accounting for the basic design components (i.e., treatment group and baseline depression severity). This suggests that some of the variance initially attributed to therapists and sites may reflect patient‐level factors captured by these variables, although a considerable amount of residual variance likely remains due to unmeasured patient characteristics, contextual influences or measurement error. Moreover, this small variance could not be explained by patients' or therapists' characteristics. On the other hand, the variance in the differential treatment effect, that is, the variance between study sites in the comparative outcome of the investigated treatments, was found to be relatively large. This relatively large variance could partially be explained by therapist characteristics, that is, by the fact the therapists differed across clinics. However, the site‐related variance in differential treatment effect remained comparatively large nonetheless and was not accounted for by any other factors measured in the present study.

The amount of heterogeneity in treatment outcome between therapists is in line with findings of previous studies. Therapist‐related variance in treatment outcome appears smaller in RCTs than in naturalistic settings (Baldwin and Imel 2013; Wampold and Owen 2021). We found an even smaller variance in treatment outcome between therapist (1.1%) than Baldwin and Imel (2013), which could be due to the highly standardized and manualized nature of the investigated treatments. The fact that this variance could not be explained by commonly investigated therapist characteristics is also in line with previous research, which has equally failed to establish therapist characteristics as robust predictors of therapy outcome (Baldwin and Imel 2013; Wampold and Owen 2021). The absence of clear explanatory factors is also unsurprising considering the already very small size of the therapist variance in treatment outcome. Second, the heterogeneity in treatment outcomes between sites of 1.7% in our study appears in line with findings in naturalistic settings (Firth et al. 2019; Mahon et al. 2023; Pybis et al. 2017) whereas the few studies on outcome heterogeneity in RCTs reported variance estimates between 3% and 9% (Bickman et al. 2011; Delgadillo et al. 2016; Vaezazizi et al. 2019). As all of these RCTs investigated the variance across a small number of study sites, it is not surprising that the estimate of the site‐related variance has a relatively large range. In sum, the small heterogeneity in treatment outcomes between therapists and sites in the present study supports the idea that randomized controlled designs can effectively control for external factors that may bias treatment outcomes. Third, our relatively large site‐related heterogeneity in differential treatment effect of 12.5% is difficult to put into context, given that previous investigations in this area are extremely scarce or absent. In the following, we discuss some possibilities to explain these findings, limitations of the present study and how they may motivate future research.

We have identified a relatively large site‐related variance in differential treatment effect that is not attributable to the patient characteristics and only partially to therapist characteristics. In previous studies, patients' socio‐economic status could explain large portion of site‐related outcome variance (Firth et al. 2019). However, in the present study, using the income of the patients as a proxy for their socio‐economic status did not explain a notable amount of variance. Differences in organizational culture could be another important explanatory factor, as indicated in the study by Falkenström et al. (2018). However, due to a lack of documentation of site characteristics and, more importantly, the limited number of sites in the present study, an empirical exploration of the sources of this heterogeneity was not possible.

Still, some components of the study design might in theory explain the heterogeneity in the differential treatment effect. Besides site characteristics, a natural hypothesis might be, that differences in the implementation of the interventions between the sites may lead to variance in the differential treatment effect. First, the flexibility in delivery could impact outcome heterogeneity (Loudon et al. 2015). This means, if some study sites were stricter than others regarding protocol adherence of their therapists, the site‐related heterogeneity in treatment outcome and in treatment effect might have increased. However, in the present RCT, the treatment implementation was supervised consistently across sites through a centralized body conducting the supervision and training of the treatments across all sites. Thus, the large variance in differential treatment effect across sites is unlikely to be related to differences in training and protocol adherence across sites.

Another potentially relevant domain concerns the potential heterogeneity in the presentation of the study. It could be that the sites framed the treatments differently during the setup of the study. If, for instance, a study site presented the RCT as investigating a novel, highly promising treatment, this might have set different expectations for patients and therapists in this site, compared to sites where the RCT was framed as performing a more neutral comparison of two active psychotherapeutic treatments. Similarly, therapists in the same study site might share their allegiance to a specific therapeutic approach and consequently favour one study treatment over the other. Outcome expectations have been shown to have a considerable impact on the treatment outcome, in a positive as well as negative direction (e.g., Constantino et al. 2021).

This aspect of an RCT is indeed difficult to standardize. While the patient information forms were consistent across sites in the current study, the verbal transmission of the study from the principal investigators and clinic leadership to the therapists and from the therapists to patients was less controlled. An additional challenge in psychotherapy research is the limited possibilities to blind the treatment. This means that therapists and often also patients know which treatment they receive. This could strengthen the bias through expectancy effects. Also, in the present trial, therapists were assigned a study treatment by the study site's principal investigator. This process could potentially amplify this bias further, if the principal investigator implicitly or explicitly considered the expertise or preferences of the therapists. Thus, differences in framing—and consequently expectations—might have affected the performance of the therapists and the patients directly and, therefore, caused a rather larger heterogeneity in the differential treatment effect.

In sum, few obvious differences between the study sites or the implementation of the interventions were present. A difference in framing of the study and consequently the related expectations about the performance of the two interventions between study sites cannot be ruled out. The possible role of expectancy effects in the site‐related heterogeneity of the differential treatment effect remains to be examined empirically in future studies. Systematically assessing and documenting site characteristics in multicentre studies, for instance, relating to the work culture or theoretical dominance of certain approaches—among many others—may provide a fertile ground for investigating these issues in future studies. It is possible that some of the site's characteristics could explain a potential heterogeneity in the differential treatment effect.

Next, it is unclear how well our findings generalize to other psychotherapy RCTs. While the present RCT was relatively large, the amount of site‐related variance is still estimated with a high degree of uncertainty. The present study was, thus, still underpowered to precisely estimate the therapist‐ and site‐related variance. More precise estimates of site‐related variance are likely to require data from about 30 study sites or more (Maas and Hox 2005). However, while our Bayesian analysis did not resolve this issue, we believe it is a strength of the analysis to quantify this uncertainty, as most previous studies that investigated site and therapist‐related outcome variance only reported traditional point estimates, without clear indications of precision. Furthermore, despite this limited precision of the estimated size of the variance, the relative size of the variances in the present study appears robust. Site‐related heterogeneity in the differential treatment effect was consistently larger than the other, more intensively studied sources of heterogeneity. Thus, it could be important to pay more attention to investigating heterogeneity in differential treatment effects in future multicentre studies. As few RCTs will include a sufficient number of sites to give precise estimates of site‐related heterogeneity, this will likely require a pooling of data. However, also for individual RCTs, estimating and controlling for site‐related variance in the differential treatment effect could be advisable. This could entail the use of three‐level multilevel models (cross‐level interaction) or including a fixed interaction term between sites and treatment group in the statistical model.

In conclusion, the present study provides evidence that therapist‐ and site‐related variation in treatment outcome is small and hence controllable through well‐designed RCTs. However, the site‐related heterogeneity in differential treatment effects appears comparatively large. It should be investigated further if this generalizes to other psychotherapy RCTs. Should this be the case, it would warrant further research into what factors explain this relatively large heterogeneity between study sites. Identified factors would provide valuable information for implementing the investigated treatments in practice. Contextual factors that impact the efficacy of certain treatments could also be considered in treatment recommendations. It might also be necessary to control for these factors through study design or in the statistical evaluation of RCTs. In the long term, routinely investigating and accounting this source of heterogeneity could improve the understanding of contextual factors on the treatment effect and increase the generalizability and replicability of psychotherapy research.

## Author Contributions

**Jonas Petter:** conceptualization, methodology, formal analysis, visualization, writing – original draft. **Lea Schumacher:** methodology, writing – review and editing. **Jette Echterhoff:** writing – review and editing. **Jan Philipp Klein:** resources, writing – review and editing. **Elisabeth Schramm:** resources, writing – review and editing. **Martin Härter:** resources, writing – review and editing. **Martin Hautzinger:** resources, writing – review and editing. **Levente Kriston:** conceptualization, methodology, writing – original draft, supervision, project administration, funding acquisition.

## Conflicts of Interest

The authors declare no conflicts of interest.

## Supporting information


**Table S1.** Overview of changes to the preregistration.
**Table S2.** Overview of the variance partition for the variance partitioning model.
**Table S3.** Overview of the variance partition for the therapist characteristics model.
**Table S4.** Overview of the variance partition for the patient characteristics model.
**Table S5.** Overview of the variance partition for the patient and therapist characteristics model.
**Table S6.** Overview of the variance partition for the extended patient and therapist characteristics model.
**Table S7.** Coefficients of the variance partitioning model.
**Table S8.** Random effects of the variance partitioning model.
**Table S9.** Coefficients of the therapist characteristics model.
**Table S10.** Random effects of the therapist characteristics model.
**Table S11.** Coefficients of the patient characteristics model.
**Table S12.** Random effects of the patient characteristics model.
**Table S13.** Coefficients of the patient and therapist characteristics model.
**Table S14.** Random effects of the patient and therapist characteristics model.
**Table S15.** Coefficients of the extended patient and therapist characteristics model.
**Table S16.** Random effects of the extended patient and therapist characteristics model.
**Table S17.** Standardized coefficients of the variance partitioning model.
**Table S18.** Standardized coefficients of the therapist characteristics model.
**Table S19.** Standardized coefficients of the patient characteristics model.
**Table S20.** Standardized coefficients of the patient and therapist characteristics model.
**Table S21.** Standardized coefficients of the extended patient and therapist characteristics model.
**Table S22.** Number of missing values in patient data.
**Table S23.** Number of missing values in therapist data.
**Table S24.** Model comparison with fit indices.

## Data Availability

The data that support the findings of this study are available on request from the corresponding author, given approval of all other authors. The data are not publicly available due to privacy and ethical restrictions. The data reported in this manuscript have been previously published and were collected as part of a RCT with the aim to compare the effectiveness of two psychotherapeutic interventions. Findings from the data collection have been reported in separate manuscripts. In total, 14 manuscripts have been published that analyse different variables of the dataset or the same variables applying different statistical methods.
