# Supplementary material for: Heterogeneity of Treatment Outcomes Across Therapists and Sites in a Randomized Multicentre Psychotherapy Trial
Source: Clin Psychol Psychother. 2025 May 21;32(3):e70087. doi: 10.1002/cpp.70087 (PMC12095096; doi:10.1002/cpp.70087)
Supplement: Supplementary file 1 — Table S1. Overview of changes to the preregistration. Table S2. Overview of the variance partition for the variance partitioning model. Table S3. Overview of the variance partition for the therapist characteristics model. Table S4. Overview of the variance partition for the patient characteristics model. Table S5. Overview of the variance partition for the patient and therapist characteristics model. Table S6. Overview of the variance partition for the extended patient and therapist characteristics model. Table S7. Coefficients of the variance partitioning model. Table S8. Random effects of the variance partitioning model. Table S9. Coefficients of the therapist characteristics model. Table S10. Random effects of the therapist characteristics model. Table S11. Coefficients of the patient characteristics model. Table S12. Random effects of the patient characteristics model. Table S13. Coefficients of the patient and therapist characteristics model. Table S14. Random effects of the patient and therapist characteristics model. Table S15. Coefficients of the extended patient and therapist characteristics model. Table S16. Random effects of the extended patient and therapist characteristics model. Table S17. Standardized coefficients of the variance partitioning model. Table S18. Standardized coefficients of the therapist characteristics model. Table S19. Standardized coefficients of the patient characteristics model. Table S20. Standardized coefficients of the patient and therapist characteristics model. Table S21. Standardized coefficients of the extended patient and therapist characteristics model. Table S22. Number of missing values in patient data. Table S23. Number of missing values in therapist data. Table S24. Model comparison with fit indices. [file CPP-32-e70087-s001.docx]

# **Supplement - Heterogeneity of Treatment Outcomes Across Therapists and Sites in a Randomized Multicenter Psychotherapy Trial**

*Jonas Petter^1^, Lea Schumacher^1^, Jette Echterhoff^1^, Jan Philipp Klein^2^, Elisabeth Schramm^3^, Martin Härter^1^,* *Martin Hautzinger^4^, Levente Kriston^1^*

^1^Department of Medical Psychology, University Medical Center Hamburg-Eppendorf, Hamburg, Germany

^2^Department of Psychiatry, Psychosomatics and Psychotherapy, University of Lübeck, Lübeck, Germany

^3^Department of Psychiatry and Psychotherapy, Medical Center, Faculty of Medicine, University of Freiburg, Freiburg, Germany

^4^Department of Psychology, Clinical Psychology, and Psychotherapy, Eberhard Karls University of Tübingen, Tübingen, Germany

**Table S1**

*Overview of Changes to the Preregistration*

| Pre-Registration Statement | Change | Justification |
| --- | --- | --- |
| “… 260 received the treatment as randomized (135 CBASP, 125 SP). These patients were clustered to receive treatment by 81 therapists…” | Only 255 patients from 79 therapists included in the analysis. | Five patients changed therapist throughout treatment. Two therapists did not complete therapy with any patient. These were excluded. |
| “We will explore the following patient characteristics as potential sources of heterogeneity in the treatment outcome: […]   - Number of physical comorbidities” | The number of physical comorbidities was not included as a predictor in the patient-characteristics variable block. | There was almost no variance in this variable. |
| “- Baseline anxiety level, measured by the anxiety scale and the phobic anxiety scale from the Brief Symptom Inventory (BSI) and the General Anxiety Disorder Assessment(GAD-7)” | Only included the GAD-7 scores. | To avoid including two measures of the same construct or incoherent combinations of the two scales. |
| “Childhood trauma, measured by the Childhood Trauma Questionnaire (CTQ) and the Early Trauma Inventory (ETI).” | Only included the CTQ scores. | To avoid including two measures of the same construct or incoherent combinations of the two scales. |

## Formal Expression of Models

The formal expression of the therapist characteristics model is given in (S1).

| $y_{ijk}=\beta_{000}+u_{00k}+u_{0jk}+(\beta_{100}+u_{10k})\cdot\text{Treatment}_{ijk}+\beta_{200}\cdot\text{BaselineDep}_{ijk}+\beta_{300}\cdot\text{TherapistAge}_{ijk}+\beta_{400}\cdot\text{TherapistGender}_{ijk}+\beta_{500}\cdot\text{GeneralExp}_{ijk}+\beta_{600}\cdot\text{AssignedExp}_{ijk}+\beta_{700}\cdot\text{AcademicBG}_{ijk}+\beta_{800}\cdot\text{ManualExp}_{ijk}+\beta_{900}\cdot\text{TherapyID}_{ijk}+e_{ijk}$ | (S1) |
| --- | --- |

Where $y_{ijk}$​ represents the outcome for patient *i*, with therapist *j*, in clinic *k*. The intercept term $\beta_{000}$​ is the overall mean, while $u_{00k}$​ is the random intercept at the clinic level. The term $u_{0jk}$​ represents the random intercept at the therapist level. The coefficient $\beta_{100}$ corresponds to the overall treatment effect, with $u_{10k}$​ representing the random slope for treatment at the clinic level. $\text{Treatment}_{ijk}$​ is the treatment indicator, and $\text{BaselineDep}_{ijk}$​ represents the baseline depression score for patient *i*. The additional terms account for therapist-level covariates: $\text{TherapistAge}_{ijk}$​, $\text{TherapistGender}_{ijk}$, $\text{GeneralExp}_{ijk}$​ (general psychotherapy experience), $\text{AssignedExp}_{ijk}$​ (experience with the assigned therapy), $\text{AcademicBG}_{ijk}$​ (academic background), $\text{ManualExp}_{ijk}$​ (experience with manualized therapy), and $\text{TherapyID}_{ijk}$​ (identification with the assigned therapy). Finally, $e_{ijk}$ is the residual error at the patient level.

Next, the formal expression of the patient characteristics model is given in (S2).

| $y_{ijk}=\beta_{000}+u_{00k}+u_{0jk}+(\beta_{100}+u_{10k})\cdot\text{Treatment}_{ijk}+\beta_{200}\cdot\text{BaselineDep}_{ijk}+\beta_{300}\cdot\text{PatientAge}_{ijk}+\beta_{400}\cdot\text{PatientGender}_{ijk}+\beta_{500}\cdot\text{BaselineAnx}_{ijk}+\beta_{600}\cdot\text{SuicidalIdeation}_{ijk}+\beta_{700}\cdot\text{SocialFunc}_{ijk}+\beta_{800}\cdot\text{Interpersonal}_{ijk}+\beta_{900}\cdot\text{Comorbidity}_{ijk}+\beta_{1000}\cdot\text{DepType}_{ijk}+\beta_{1100}\cdot\text{Trauma}_{ijk}+\beta_{1200}\cdot\text{TxHistory}_{ijk}+\beta_{1300}\cdot\text{PsychPref}_{ijk}+\beta_{1400}\cdot\text{Income}_{ijk}+e_{ijk}$ | (S2) |
| --- | --- |

Where $y_{ijk}$​ represents the outcome for patient *i*, with therapist *j*, in clinic *k*. The intercept term $\beta_{000}$​ is the overall mean, while $u_{00k}$​ is the random intercept at the clinic level. The term $u_{0jk}$​ represents the random intercept at the therapist level. The coefficient $\beta_{100}$ corresponds to the overall treatment effect, with $u_{10k}$​ representing the random slope for treatment at the clinic level. $\text{Treatment}_{ijk}$​ is the treatment indicator, and $\text{BaselineDep}_{ijk}$​ represents the baseline depression score for patient *i*. The remaining terms represent additional patient-level covariates: $\text{PatientAge}_{ijk}$​, $\text{PatientGender}_{ijk}$​, $\text{BaselineAnx}_{ijk}$​ (anxiety severity at baseline), $\text{SuicidalIdeation}_{ijk}$​, $\text{SocialFunc}_{ijk}$​ (social functioning), $\text{Interpersonal}_{ijk}$​ (interpersonal problems), $\text{Comorbidity}_{ijk}$​ (psychological comorbidities), $\text{DepType}_{ijk}$​ (type of depression), $\text{Trauma}_{ijk}$​ (childhood trauma), $\text{TxHistory}_{ijk}$​ (previous treatment history), $\text{PsychPref}_{ijk}$​ (preference for psychotherapy over medication), and $\text{Income}_{ijk}$​ (income level).

Next, the formal expression of the patient and therapist characteristics model is given in (S3).

| $y_{ijk}=\beta_{000}+u_{00k}+u_{0jk}+(\beta_{100}+u_{10k})\cdot\text{Treatment}_{ijk}+\beta_{200}\cdot\text{BaselineDep}_{ijk}+\beta_{300}\cdot\text{TherapistAge}_{ijk}+\beta_{400}\cdot\text{TherapistGender}_{ijk}+\beta_{500}\cdot\text{TherapyExp}_{ijk}+\beta_{600}\cdot\text{TherapyCases}_{ijk}+\beta_{700}\cdot\text{TherapistBG}_{ijk}+\beta_{800}\cdot\text{ManualExp}_{ijk}+\beta_{900}\cdot\text{TherapyID}_{ijk}+\beta_{1000}\cdot\text{PatientAge}_{ijk}+\beta_{1100}\cdot\text{PatientGender}_{ijk}+\beta_{1200}\cdot\text{BaselineAnx}_{ijk}+\beta_{1300}\cdot\text{SuicidalIdeation}_{ijk}+\beta_{1400}\cdot\text{SocialFunc}_{ijk}+\beta_{1500}\cdot\text{Interpersonal}_{ijk}+\beta_{1600}\cdot\text{Comorbidity}_{ijk}+\beta_{1700}\cdot\text{DepType}_{ijk}+\beta_{1800}\cdot\text{Trauma}_{ijk}+\beta_{1900}\cdot\text{TxHistory}_{ijk}+\beta_{2000}\cdot\text{PsychPref}_{ijk}+\beta_{2100}\cdot\text{Income}_{ijk}+e_{ijk}$ | (S3) |
| --- | --- |

Where $y_{ijk}$​ represents the outcome for patient *i*, with therapist *j*, in clinic *k*. The random intercepts $u_{00k}$​ and $u_{0jk}$​ account for clinic and therapist clustering, respectively, and $u_{10k}$​ is the random slope for treatment across clinics. The fixed effects include the treatment condition, baseline depression, and therapist- and patient-level covariates. All other predictors are as described above.

Lastly, the formal expression of the extended patient and therapist characteristics model is given in (S4).

| $y_{ijk}=\beta_{000}+u_{00k}+u_{0jk}+(\beta_{100}+u_{10k})\cdot\text{Treatment}_{ijk}+\beta_{200}\cdot\text{BaselineDep}_{ijk}+\beta_{300}\cdot\text{TherapistAge}_{ijk}+\beta_{400}\cdot\text{TherapistGender}_{ijk}+\beta_{500}\cdot\text{TherapyExp}_{ijk}+\beta_{600}\cdot\text{TherapyCases}_{ijk}+\beta_{700}\cdot\text{TherapistBG}_{ijk}+\beta_{800}\cdot\text{ManualExp}_{ijk}+\beta_{900}\cdot\text{TherapyID}_{ijk}+\beta_{1000}\cdot\text{PatientAge}_{ijk}+\beta_{1100}\cdot\text{PatientGender}_{ijk}+\beta_{1200}\cdot\text{BaselineAnx}_{ijk}+\beta_{1300}\cdot\text{SuicidalIdeation}_{ijk}+\beta_{1400}\cdot\text{SocialFunc}_{ijk}+\beta_{1500}\cdot\text{Interpersonal}_{ijk}+\beta_{1600}\cdot\text{Comorbidity}_{ijk}+\beta_{1700}\cdot\text{DepType}_{ijk}+\beta_{1800}\cdot\text{Trauma}_{ijk}+\beta_{1900}\cdot\text{TxHistory}_{ijk}+\beta_{2000}\cdot\text{PsychPref}_{ijk}+\beta_{2100}\cdot\text{Income}_{ijk}+\beta_{2200}\cdot\text{AgeSimilarity}_{ijk}+\beta_{2300}\cdot\text{GenderMatch}_{ijk}+e_{ijk}$ | (S4) |
| --- | --- |

Where $y_{ijk}$​ represents the outcome for patient *i*, with therapist *j*, in clinic *k*. The random intercepts $u_{00k}$​ and $u_{0jk}$​ account for clinic and therapist clustering, respectively, and $u_{10k}$​ is the random slope for treatment across clinics. The fixed effects include the treatment condition, baseline depression, and therapist- and patient-level covariates. In addition, two dyadic variables are included: $\text{AgeSimilarity}_{ijk}$ (patient–therapist age similarity) and ​$\text{GenderMatch}_{ijk}$ (patient–therapist gender congruence). All other predictors are as described above.

## Secondary Outcome (Number of Therapy Sessions) Output

**Table S2**

*Overview of the Variance Partition for the Variance Partitioning Model*

| Variance Source | Variance | VPC |
| --- | --- | --- |
| Therapist intercept | 0.4046356 | 0.780435 |
| Clinic intercept | 0.7252790 | 1.398871 |
| Clinic slope | 5.9478041 | 11.471741 |
| Residual | 44.7697233 | 86.348953 |
| Total | 51.8474420 | 100.000000 |

**Table S3**

*Overview of the Variance Partition for the Therapist Characteristics Model*

| Variance Source | Variance | VPC |
| --- | --- | --- |
| Therapist intercept | 0.5059911 | 0.9863689 |
| Clinic intercept | 0.7488351 | 1.4597641 |
| Clinic slope | 4.9138459 | 9.5789531 |
| Residual | 45.1296891 | 87.9749140 |
| Total | 51.2983611 | 100.0000000 |

**Table S4**

*Overview of the Variance Partition for the Patient Characteristics Model*

| Variance Source | Variance | VPC |
| --- | --- | --- |
| Therapist intercept | 0.5165645 | 0.9600037 |
| Clinic intercept | 0.8218029 | 1.5272709 |
| Clinic slope | 7.1645385 | 13.3148600 |
| Residual | 45.3056842 | 84.1978654 |
| Total | 53.8085901 | 100.0000000 |

**Table S5**

*Overview of the Variance Partition for the Patient and Therapist Characteristics Model*

| Variance Source | Variance | VPC |
| --- | --- | --- |
| Therapist intercept | 0.6476203 | 1.217908 |
| Clinic intercept | 0.8841147 | 1.662657 |
| Clinic slope | 6.0792175 | 11.432516 |
| Residual | 45.5638472 | 85.686918 |
| Total | 53.1747997 | 100.000000 |

**Table S6**

*Overview of the Variance Partition for the Extended Patient and Therapist Characteristics Model*

| Variance Source | Variance | VPC |
| --- | --- | --- |
| Therapist intercept | 0.7282083 | 1.375934 |
| Clinic intercept | 1.0666666 | 2.015444 |
| Clinic slope | 6.1992345 | 11.713323 |
| Residual | 44.9305336 | 84.895298 |
| Total | 52.9246430 | 100.000000 |

## Full Model Output (Outcome: Depression Reduction)

**Table S7**

*Coefficients of the Variance Partitioning Model*

| Predictor | Estimate | Est. Error | 95% CI LB | 95% CI UB |
| --- | --- | --- | --- | --- |
| Intercept | 8.1087603 | 0.7512075 | 6.6476374 | 9.5817819 |
| Treatment Group | 2.6481998 | 1.7201273 | -0.8122631 | 6.1097049 |
| Baseline Depression | 0.3712419 | 0.0985515 | 0.1738855 | 0.5640499 |

**Table S8**

*Random Effects of the Variance Partitioning Model*

| Parameter | Estimate | Est. Error | | 95% CI LB | | 95% CI UB | |  |
| --- | --- | --- | --- | --- | --- | --- | --- | --- |
| SD(Therapist Intercept) | 1.06 | 0.76 | | 0.05 | | 2.81 | |  |
| SD(Clinic Intercept) | 1.2186380 | | 0.8568013 | | 0.0609146 | | 3.316786 | |
| SD(Clinic Slope) | 3.4086339 | | 1.8792469 | | 0.3461087 | | 7.645256 | |
| Cor(Clinic Intercept, Clinic Slope) | 0.3774949 | | 0.5124196 | | -0.8235252 | | 0.983680 | |

**Table S9**

*Coefficients of the Therapist Characteristics Model*

| Predictor | Estimate | Est. Error | 95% CI LB | 95% CI UB |
| --- | --- | --- | --- | --- |
| Intercept | 8.0438071 | 0.8253931 | 6.3938025 | 9.6223029 |
| Treatment Group | 1.6529794 | 1.9677717 | -2.2103814 | 5.7113330 |
| Baseline Depression | 0.3694682 | 0.1031267 | 0.1679532 | 0.5734270 |
| Therapist Age | -0.1063503 | 0.1050262 | -0.3097438 | 0.1079873 |
| Therapist Gender | -0.1094256 | 1.4502548 | -2.9996038 | 2.7089827 |
| Academic Background | 1.0185831 | 1.6882013 | -2.1638994 | 4.4576104 |
| Clinical Experience | 0.0150920 | 0.0147280 | -0.0143797 | 0.0437937 |
| Experience Manualized Therapy | 1.6301060 | 1.3712246 | -1.0685795 | 4.3453138 |
| Experience Assigned Treatment | -0.1182481 | 0.1242498 | -0.3641451 | 0.1210170 |
| Attitude Assigned Treatment | 0.2976533 | 0.4211324 | -0.5387150 | 1.1176982 |

**Table S10**

*Random Effects of the Therapist Characteristics Model*

| Parameter | Estimate | Est. Error | | 95% CI LB | | 95% CI UB | | |
| --- | --- | --- | --- | --- | --- | --- | --- | --- |
| SD(Therapist Intercept) | 1.14 | 0.80 | | 0.05 | | 2.99 | | |
| SD(Clinic Intercept) | 1.3954635 | | 0.9999258 | | 0.0747028 | | 3.7727140 |  |
| SD(Clinic Slope) | 2.8011879 | | 1.8284792 | | 0.1440961 | | 7.0315454 |  |
| Cor(Clinic Intercept, Clinic Slope) | 0.2746934 | | 0.5359890 | | -0.8582206 | | 0.9801711 |  |

**Table S11**

*Coefficients of the Patient Characteristics Model*

| Predictor | Estimate | Est. Error | 95% CI LB | 95% CI UB |
| --- | --- | --- | --- | --- |
| Intercept | 8.0465039 | 0.8219455 | 6.4167154 | 9.7151077 |
| Treatment Group | 1.2719968 | 1.6448505 | -2.0226932 | 4.5805770 |
| Baseline Depression | 0.5674686 | 0.1102041 | 0.3616095 | 0.7909215 |
| Patient Gender | 0.1324251 | 1.1321921 | -2.0766956 | 2.3940539 |
| Treatment Preference | -0.9879368 | 0.7676650 | -2.4912189 | 0.5141250 |
| Depression Type | -1.0965076 | 0.6775933 | -2.4553610 | 0.2333012 |
| Income | -0.2258877 | 0.0926427 | -0.4108121 | -0.0453180 |
| Patient Age | -0.0956433 | 0.0471068 | -0.1856314 | -0.0047945 |
| Suicidality | -0.1762011 | 0.0810126 | -0.3353149 | -0.0206487 |
| Social Functioning | -0.0590405 | 0.0984978 | -0.2531530 | 0.1280436 |
| Interpersonal Problems | -0.1587072 | 0.1671267 | -0.4934478 | 0.1645758 |
| Childhood Trauma | 0.0183328 | 0.0374287 | -0.0557778 | 0.0908665 |
| Baseline Anxiety | -0.3392790 | 0.1286102 | -0.5856452 | -0.0867476 |
| Psychological Comorbidities | -0.2934503 | 0.8611772 | -2.0192244 | 1.3976674 |
| Treatment History | -1.0607929 | 0.5018359 | -2.0420758 | -0.0990301 |

**Table S12**

*Random Effects of the Patient Characteristics Model*

| Parameter | Estimate | Est. Error | | 95% CI LB | | 95% CI UB | | |
| --- | --- | --- | --- | --- | --- | --- | --- | --- |
| SD(Therapist Intercept) | 0.95 | 0.69 | | 0.05 | | 2.57 | | |
| SD(Clinic Intercept) | 1.5835026 | | 0.9224747 | | 0.1412005 | | 3.7862762 |  |
| SD(Clinic Slope) | 3.1861145 | | 1.7729388 | | 0.3148010 | | 7.2938773 |  |
| Cor(Clinic Intercept, Clinic Slope) | 0.4932454 | | 0.4522747 | | -0.6498278 | | 0.9859462 |  |

**Table S13**

*Coefficients of the Patient and Therapist Characteristics Model*

| Predictor | Estimate | Est. Error | 95% CI LB | 95% CI UB |
| --- | --- | --- | --- | --- |
| Intercept | 8.0568731 | 0.9192245 | 6.2323142 | 9.9121589 |
| Treatment Group | 0.6948008 | 1.8671960 | -2.7973162 | 4.6262083 |
| Baseline Depression | 0.5762510 | 0.1108431 | 0.3552249 | 0.7925542 |
| Patient Gender | 0.1439972 | 1.1478801 | -2.1533304 | 2.3405667 |
| Treatment Preference | -0.9072246 | 0.8061363 | -2.5002479 | 0.6195642 |
| Depression Type | -1.0698009 | 0.7236411 | -2.4837781 | 0.3692800 |
| Income | -0.2580117 | 0.0951555 | -0.4482343 | -0.0772153 |
| Patient Age | -0.0889544 | 0.0479287 | -0.1817705 | 0.0055959 |
| Suicidality | -0.1693364 | 0.0826213 | -0.3346449 | -0.0098481 |
| Social Functioning | -0.0465559 | 0.1047251 | -0.2556818 | 0.1587027 |
| Interpersonal Problems | -0.1636104 | 0.1788021 | -0.5157634 | 0.1755828 |
| Childhood Trauma | 0.0167530 | 0.0376787 | -0.0556258 | 0.0908816 |
| Baseline Anxiety | -0.3248155 | 0.1343863 | -0.5955380 | -0.0656527 |
| Psychological Comorbidities | -0.1861315 | 0.8865219 | -1.9320318 | 1.5582877 |
| Treatment History | -1.1128996 | 0.5202564 | -2.1506693 | -0.0927629 |
| Therapist Age | -0.1071017 | 0.0996902 | -0.3051329 | 0.0834179 |
| Therapist Gender | -0.5359559 | 1.3802122 | -3.1290417 | 2.1710542 |
| Academic Background | 0.5269708 | 1.6909336 | -2.7867372 | 3.8613010 |
| Clinical Experience | 0.0152112 | 0.0134780 | -0.0116186 | 0.0419211 |
| Experience Manualized Therapy | 1.7092413 | 1.3156645 | -0.8310782 | 4.2410791 |
| Experience Assigned Treatment | -0.1049278 | 0.1231334 | -0.3375011 | 0.1373452 |
| Attitude Assigned Treatment | 0.0689655 | 0.4000369 | -0.7136358 | 0.8825494 |

**Table S14**

*Random Effects of the Patient and Therapist Characteristics Model*

| Parameter | Estimate | Est. Error | | 95% CI LB | | 95% CI UB | | |
| --- | --- | --- | --- | --- | --- | --- | --- | --- |
| SD(Therapist Intercept) | 1.03 | 0.76 | | 0.05 | | 2.80 | | |
| SD(Clinic Intercept) | 1.9089757 | | 1.0888566 | | 0.2087341 | | 4.5359447 |  |
| SD(Clinic Slope) | 2.8182739 | | 1.7184147 | | 0.1981536 | | 6.8602965 |  |
| Cor(Clinic Intercept, Clinic Slope) | 0.4197244 | | 0.4885712 | | -0.7629680 | | 0.9839951 |  |

**Table S15**

*Coefficients of the Extended Patient and Therapist Characteristics Model*

| Predictor | Estimate | Est. Error | 95% CI LB | 95% CI UB |
| --- | --- | --- | --- | --- |
| Intercept | 8.0854177 | 0.9574223 | 6.3200170 | 10.0123508 |
| Treatment Group | 0.7866927 | 1.9096545 | -2.5755132 | 4.6495208 |
| Baseline Depression | 0.5768247 | 0.1127603 | 0.3566941 | 0.8024544 |
| Patient Gender | 0.3260703 | 1.2645896 | -2.1346867 | 2.8371953 |
| Treatment Preference | -0.8936090 | 0.8203951 | -2.5382328 | 0.7218365 |
| Depression Type | -1.0925383 | 0.7180662 | -2.4984717 | 0.3149794 |
| Income | -0.2596126 | 0.0972815 | -0.4532532 | -0.0670781 |
| Patient Age | -0.0829728 | 0.2155865 | -0.4996211 | 0.3567554 |
| Suicidality | -0.1654714 | 0.0842345 | -0.3286969 | -0.0026357 |
| Social Functioning | -0.0497661 | 0.1014167 | -0.2477331 | 0.1489633 |
| Interpersonal Problems | -0.1734747 | 0.1756029 | -0.5233947 | 0.1833423 |
| Childhood Trauma | 0.0188672 | 0.0383329 | -0.0562623 | 0.0948485 |
| Baseline Anxiety | -0.3272207 | 0.1362872 | -0.5955519 | -0.0637052 |
| Psychological Comorbidities | -0.2007007 | 0.8891806 | -1.9250765 | 1.5003900 |
| Treatment History | -1.1191948 | 0.5132042 | -2.1146370 | -0.0986932 |
| Therapist Age | -0.1142383 | 0.2300114 | -0.5823701 | 0.3323803 |
| Therapist Gender | -0.3802195 | 1.3650015 | -3.0128412 | 2.4084188 |
| Academic Background | 0.6183324 | 1.7107596 | -2.6672323 | 4.0729101 |
| Clinical Experience | 0.0151167 | 0.0141782 | -0.0127467 | 0.0423146 |
| Experience Manualized Therapy | 1.7844626 | 1.3437764 | -0.8377551 | 4.3977138 |
| Experience Assigned Treatment | -0.1127063 | 0.1253941 | -0.3608539 | 0.1367630 |
| Attitude Assigned Treatment | 0.0456911 | 0.4029873 | -0.7261138 | 0.8459322 |
| Congruence Gender | -0.4921744 | 1.2902180 | -3.0393051 | 1.9966264 |
| Congruence Age | 0.0070117 | 0.2119474 | -0.4074061 | 0.4280444 |

**Table S16**

*Random Effects of the Extended Patient and Therapist Characteristics Model*

| Parameter | Estimate | Est. Error | | 95% CI LB | | 95% CI UB | | |
| --- | --- | --- | --- | --- | --- | --- | --- | --- |
| SD(Therapist Intercept) | 1.02 | 0.74 | | 0.04 | | 2.76 | | |
| SD(Clinic Intercept) | 1.909015 | | 1.1241977 | | 0.1626680 | | 4.5563778 |  |
| SD(Clinic Slope) | 2.817677 | | 1.8302700 | | 0.1716928 | | 7.3513100 |  |
| Cor(Clinic Intercept, Clinic Slope) | 0.410341 | | 0.4876211 | | -0.7727774 | | 0.9851861 |  |

## Standardized Regression Coefficients (Outcome: Depression Reduction)

**Table S17**

*Standardized Coefficients of the Variance Partitioning Model*

| Predictor | Estimate | 95% CI LB | 95% CI UB |
| --- | --- | --- | --- |
| Intercept | 0.0012655 | -0.1830913 | 0.1617304 |
| Treatment Group | 0.1431750 | -0.0521518 | 0.3491833 |
| Baseline Depression | 0.2339858 | 0.1104241 | 0.3493388 |

**Table S18**

*Standardized Coefficients of the Therapist Characteristics Model*

| Predictor | Estimate | 95% CI LB | 95% CI UB |
| --- | --- | --- | --- |
| Intercept | -0.0053945 | -0.2021100 | 0.1679938 |
| Treatment Group | 0.0992203 | -0.0882588 | 0.3179249 |
| Baseline Depression | 0.2250925 | 0.1027213 | 0.3516395 |
| Therapist Age | -0.0496773 | -0.2086224 | 0.1063413 |
| Therapist Gender | -0.0018157 | -0.1483079 | 0.1398554 |
| Academic Background | 0.0498206 | -0.1057930 | 0.2091155 |
| Clinical Experience | 0.0347981 | -0.0800112 | 0.1474072 |
| Experience Manualized Therapy | 0.0952055 | -0.0527634 | 0.2409062 |
| Experience Assigned Treatment | -0.0790131 | -0.2320705 | 0.0788831 |
| Attitude Assigned Treatment | 0.0567407 | -0.1173051 | 0.2305907 |

**Table S19**

*Standardized Coefficients of the Patient Characteristics Model*

| Predictor | Estimate | 95% CI LB | 95% CI UB |
| --- | --- | --- | --- |
| Intercept | -0.0083429 | -0.2098555 | 0.1860897 |
| Treatment Group | 0.0615767 | -0.1254440 | 0.2538611 |
| Baseline Depression | 0.3553793 | 0.2257225 | 0.4927666 |
| Patient Gender | 0.0066717 | -0.1101021 | 0.1229302 |
| Treatment Preference | -0.0730356 | -0.1925408 | 0.0421273 |
| Depression Type | -0.0936310 | -0.2095637 | 0.0213702 |
| Income | -0.1497386 | -0.2665486 | -0.0328453 |
| Patient Age | -0.1193677 | -0.2429926 | -0.0033010 |
| Suicidality | -0.1424948 | -0.2658084 | -0.0171783 |
| Social Functioning | -0.0437247 | -0.1824246 | 0.0959508 |
| Interpersonal Problems | -0.0682464 | -0.2150617 | 0.0729594 |
| Childhood Trauma | 0.0306285 | -0.0917511 | 0.1495003 |
| Baseline Anxiety | -0.1697303 | -0.2984535 | -0.0402113 |
| Psychological Comorbidities | -0.0220120 | -0.1425477 | 0.0957288 |
| Treatment History | -0.1278377 | -0.2427334 | -0.0120776 |

**Table S20**

*Standardized Coefficients of the Patient and Therapist Characteristics Model*

| Predictor | Estimate | 95% CI LB | 95% CI UB |
| --- | --- | --- | --- |
| Intercept | -0.0066014 | -0.2134453 | 0.1914209 |
| Treatment Group | 0.0475448 | -0.1529380 | 0.2641941 |
| Baseline Depression | 0.3570906 | 0.2126317 | 0.4966417 |
| Patient Gender | 0.0093210 | -0.1084677 | 0.1276577 |
| Treatment Preference | -0.0672247 | -0.1893122 | 0.0537505 |
| Depression Type | -0.0933770 | -0.2144915 | 0.0290147 |
| Income | -0.1650092 | -0.2894457 | -0.0412729 |
| Patient Age | -0.1145728 | -0.2385352 | 0.0082803 |
| Suicidality | -0.1312636 | -0.2605635 | -0.0059469 |
| Social Functioning | -0.0290271 | -0.1700756 | 0.1107551 |
| Interpersonal Problems | -0.0702113 | -0.2041028 | 0.0729533 |
| Childhood Trauma | 0.0304265 | -0.0871648 | 0.1543885 |
| Baseline Anxiety | -0.1650472 | -0.3068488 | -0.0330217 |
| Psychological Comorbidities | -0.0205809 | -0.1435957 | 0.0972240 |
| Treatment History | -0.1329405 | -0.2582985 | -0.0110496 |
| Therapist Age | -0.0543851 | -0.2142353 | 0.1096880 |
| Therapist Gender | -0.0249265 | -0.1504967 | 0.1040620 |
| Academic Background | 0.0251023 | -0.1265563 | 0.1837608 |
| Clinical Experience | 0.0374858 | -0.0783870 | 0.1555427 |
| Experience Manualized Therapy | 0.0951053 | -0.0370135 | 0.2431699 |
| Experience Assigned Treatment | -0.0702765 | -0.2219805 | 0.0799861 |
| Attitude Assigned Treatment | 0.0128348 | -0.1525724 | 0.1786394 |

**Table S21**

*Standardized Coefficients of the Extended Patient and Therapist Characteristics Model*

| Predictor | Estimate | 95% CI LB | 95% CI UB |
| --- | --- | --- | --- |
| Intercept | -0.0094057 | -0.2129348 | 0.1817714 |
| Treatment Group | 0.0486969 | -0.1472937 | 0.2568767 |
| Baseline Depression | 0.3593312 | 0.2180279 | 0.4933686 |
| Patient Gender | 0.0200020 | -0.1044479 | 0.1500271 |
| Treatment Preference | -0.0674769 | -0.1886361 | 0.0556070 |
| Depression Type | -0.0926844 | -0.2190515 | 0.0301685 |
| Income | -0.1689926 | -0.2895517 | -0.0443209 |
| Patient Age | -0.0925417 | -0.4727345 | 0.2697150 |
| Suicidality | -0.1296667 | -0.2616631 | -0.0031485 |
| Social Functioning | -0.0327941 | -0.1797392 | 0.1123436 |
| Interpersonal Problems | -0.0710138 | -0.2095412 | 0.0750288 |
| Childhood Trauma | 0.0328859 | -0.0957223 | 0.1550008 |
| Baseline Anxiety | -0.1685222 | -0.3023346 | -0.0382149 |
| Psychological Comorbidities | -0.0209002 | -0.1430932 | 0.0981429 |
| Treatment History | -0.1358999 | -0.2576085 | -0.0162134 |
| Therapist Age | -0.0664189 | -0.3666922 | 0.2396999 |
| Therapist Gender | -0.0169945 | -0.1558899 | 0.1219152 |
| Academic Background | 0.0272145 | -0.1208236 | 0.1858357 |
| Clinical Experience | 0.0367096 | -0.0770286 | 0.1489677 |
| Experience Manualized Therapy | 0.0983457 | -0.0400283 | 0.2446099 |
| Experience Assigned Treatment | -0.0731473 | -0.2235791 | 0.0798461 |
| Attitude Assigned Treatment | 0.0073019 | -0.1656444 | 0.1756693 |
| Congruence Gender | -0.0308439 | -0.1654788 | 0.1076703 |
| Congruence Age | 0.0242051 | -0.4215172 | 0.4531131 |

**Table S22**

*Number of Missing Values in Patient Data*

| Variables | Missing values |
| --- | --- |
| Age | 0 |
| Gender | 0 |
| Treatment preference | 6 |
| Income | 0 |
| Depression baseline | 0 |
| Depression outcome | 25 |
| Anxiety Baseline | 7 |
| Suicidality | 14 |
| Social functioning | 7 |
| Interpersonal problems | 6 |
| Childhood trauma | 8 |
| Depression type | 8 |
| Psychological comorbidities | 0 |
| Treatment history | 0 |

**Table S23**

*Number of Missing Values in Therapist Data*

| Variables | Missing values |
| --- | --- |
| Age | 0 |
| Gender | 0 |
| Academic background | 0 |
| Clinical experience | 0 |
| Any previous experience with manualized treatments | 0 |
| Previous experience with assigned treatment | 0 |
| Assigned treatment attitude | 0 |

## Model Comparison

**Table S24**

*Model Comparison with Fit Indices*

| **Model** | **WAIC** | **WAIC SE** | **LOOIC** | **LOOIC SE** |
| --- | --- | --- | --- | --- |
| Variance partitioning model | 1843.19 | 20.02 | 1843.45 | 20.05 |
| Therapist characteristics model | 1852.17 | 19.66 | 1852.62 | 19.70 |
| Patient characteristics model | 1819.61 | 22.08 | 1820.35 | 22.16 |
| Patient and therapist characteristics model | 1827.96 | 21.39 | 1829.15 | 21.48 |
| Extended patient and therapist characteristics model | 1828.81 | 21.28 | 1830.16 | 21.39 |
|  |  |  |  |  |
